# Serum dipeptidyl peptidase-4 activity and progranulin level in polycystic ovary syndrome patients

**DOI:** 10.22088/cjim.13.1.70

**Published:** 2022

**Authors:** Maryam Abolghasemi, Soleiman Mahjoub, Sedighe Esmaeilzadeh

**Affiliations:** 1 Student Research Committee, Babol University of Medical Sciences, Babol, Iran; 2 Department of Clinical Biochemistry, School of Medicine, Babol University of Medical Sciences, Babol, Iran; 3 Infertility and Reproductive Health Research Center, Health Research Institute, Babol University of Medical Sciences, Babol, Mazandaran, Iran

**Keywords:** Polycystic ovary syndrome, Dipeptidyl peptidase-4, Progranulin, Adipokine, Insulin resistance

## Abstract

**Background:**

Evidence showed that abnormal alteration of adipokines level may perform a key role in polycystic ovary syndrome (PCOS) pathogenesis. Dipeptidyl peptidase-4 (DPP4) and progranulin (PGRN) are two novel adipokines related to insulin resistance (IR). Thus, we aimed to determine the serum DPP4 activity and PGRN level in PCOS patients with and without IR, and non-PCOS women.

**Methods:**

Ninety women were recruited in the present study including 60 PCOS patients (divided into two groups of 30 IR and 30 non-IR) and 30 non-PCOS women. Serum levels of insulin, fasting blood glucose, PGRN, and DPP4 activity were measured, and IR indices were calculated.

**Results:**

DPP4 activity was significantly higher in PCOS-IR and PCOS-NIR patients than non-PCOS women (p<0.001, P=0.011, respectively), whereas no significant variation was detected between two groups of PCOS subjects. There was no significant difference in the level of PGRN in the three groups of the present study.

**Conclusion:**

The present study suggests that increasing DPP4 activity may be associated with PCOS.

Polycystic ovary syndrome (PCOS) is identified as the most frequent endocrine and metabolic disorder that occurs in nearly 5–10% of women of reproductive age (1). Polycystic ovaries, hyperandrogenism, and anovulation are the characteristics of this heterogeneous syndrome. Insulin resistance (IR) plays a serious impact on the development of this syndrome and can lead to reproductive abnormalities (2). Metabolic dysfunctions, particularly central adiposity, are linked to PCOS. Interestingly, the alteration of adipose tissue function takes part in the PCOS progression. Adipose tissue in the role of an endocrine organ affects various processes like glucose and lipid metabolism, and reproduction by secreting a broad spectrum of adipokines (3-5). Dipeptidyl peptidase-4 (DPP4 or CD26) in the role of a serine protease is expressed by several cells. This glycoprotein has two patterns: a transmembrane protein and a soluble pattern (6). Soluble DPP4 is a novel adipokine that partakes in the degradation of incretion hormones, indicating the momentous impact of DPP4 on metabolism (7). DPP4 was found to decline the insulin signaling pathways in various cells, including adipocytes and hepatocytes (8, 9). The results of recent investigations regarding the relationship between the activity of this enzyme and PCOS have not been conclusive. 

Although a study found that abnormal DPP4 activity was linked to the metabolic imbalance in PCOS, other studies did not show any association (10-12). Another adipokine that may contribute to PCOS etiopathogenesis is progranulin (PGRN) (13). This secreted glycoprotein is expressed by different cells and contributes to multiple processes such as cell growth, embryogenesis, inflammation, and diabetes (14). PGRN has been recognized as a momentous adipokine involved in IR and obesity (15). This adipokine in adipose tissue activates insulin insensitivity and obesity via the enhancement of the level of interleukin 6 (IL-6) (16). The studies focusing on the involvement of PGRN in the etiopathogenesis of PCOS are inadequate. In the light of a recent investigation, the reverse association between follicular fluid values of PGRN and the number of retrieved oocytes in PCOS cases have proposed that this protein may perform an impact on the oocyte development (13). 

So far, no study has perused these adipokines levels in two groups of PCOS with and without IR. Moreover, there are inconsistent results regarding DPP4 activity in PCOS cases. Therefore, given the importance of IR in the development of PCOS, our study aimed to explore DPP4 activity and PGRN concentration in the serum of PCOS cases with and without IR and non-PCOS women.

## Methods

**Participants: **Ninety women were enlisted in the present case-control study who attended the Infertility and Health Reproductive Research Center of Babol University of Medical Sciences. Sixty women were confirmed as PCOS cases and 30 non-PCOS women with regular menstrual cycles and normal ovarian were enrolled in the study as the age and body mass index (BMI) matched control individuals. The control group was selected from the infertile patients with the cause of male factor and unexplained infertility. The PCOS diagnosis was dependent upon the Rotterdam criteria. According to these criteria, at least two of three of the following characteristics are required for the diagnosis of PCOS: oligo- or anovulation, hyperandrogenism, and polycystic ovaries (17). PCOS patients were stratified into two groups based on IR: 30 PCOS women with IR (PCOS-IR) and 30 PCOS women without IR (PCOS-NIR). Informed consent was gained from all individuals included in the study and the approval was obtained from the Ethics Committee of Babol University of Medical Sciences (IR.MUBABOL.HIR.REC.1397.124). The exclusion criteria consisted of endocrine disorders, liver disease, systemic inflammatory disease, use of oral contraceptives, insulin sensitizers, and the use of obesity drugs. The demographic features of patients such as age, weight, and height were recorded. The BMI and body fat % were calculated by following formulas; BMI = Weight (kg)/ Height^2^ (m^2^); body fat % = (BMI (kg/m^2^) ×1.2) + (0.23×age (year)) – (G×10.8) – 5.4; the value of G is equal to one for men and is equal to zero for women.

**Laboratory measurements: **After 8-10 hours of fasting, blood samples (5 mL) were taken from each person to evaluate the serum values of fasting blood glucose (FBG), fasting insulin (FIN), PGRN, and DPP4 activity. The colorimetric glucose oxidase method (Pars Azmoon, Iran) was used for the analysis of the concentration of FBG (mg/dl). The serum level of FIN (μIU/ml) was analyzed by ELISA using a commercial kit (Demeditec Diagnostics GmbH, Germany). DPP4 activity was examined by the colorimetric method that is based on the conversion of glycyl-prolyl-p-nitroaniline hydrochloride (Gly-Pro-pNA, Santa Cruz Biotechnology, Dallas, TX). Hence, 10 μl of serum samples were mixed to 190 μL of 0.5 mM Gly-Pro-pNA in 50 mM Tris buffer pH 8.3 (Merck). The activity of DPP4 was detected kinetically at 37 °C every minute for 10 minutes at 405 nm by the measurement of the velocity of p-nitroaniline release from Gly-Pro-pNA (18). The DPP4 activity was reported as p-nitroaniline (μM)/mL/min. PGRN concentration was measured by a commercially available ELISA kit (Human ELISA kit, Crystal Day Biotech, Shanghai, China), based on the manufacturer’s instructions. 

**Criteria for the identification of IR: **IR status was determined according to IR indices including the homeostatic model assessment (HOMA)-IR and the quantitative insulin sensitivity check index (QUICKI). PCOS subjects with HOMA-IR ≥ 2.5 and QUICKI ≤ 0.333 were identified as IR group (19, 20). The calculation of the above mentioned IR indices was based on these formulas:

HOMA= [Fasting insulin (μIU/ml) × Fasting glucose (mg/dl)]/405

QUICKI=1/ [log fasting insulin (μIU/ml) + log fasting glucose (mg/dl)] 

**Statistical analysis**

In this study, the Kolmogorov-Smirnov normality test was done for the evaluation of variable distribution. Continuous variables are reported as the mean and standard error (SE). For the comparison of normal data, one-way analysis of variance (one-way ANOVA) was used and for the comparison of data with non-normal distribution, the Kruskal-Wallis test was used. The diagnostic accuracies of these adipokines were recognized by the receiver-operating characteristic (ROC) curve analysis and the area under the curve (AUC). Whole analyses were done by SPSS software Version 23 and a p-value < 0.05 was the level of statistical significance. 

## Results

The demographic characteristics and biochemical data of the three groups are exhibited in table 1. The mean DPP4 serum activity was significantly higher in PCOS-IR (p<0.001) and PCOS-NIR (P=0.011) patients compared to the control group. Of note, DPP4 activity was higher in PCOS-IR patients than PCOS-NIR patients, whereas this variation was not statistically significant. The mean concentration of PGRN was found to be increased in PCOS-IR and PCOS-NIR patients than the control group; however, this difference was not significant. The significant difference was not found in age, weight, BMI, and body fat % among the three groups. The cases of PCOS-IR had significantly higher levels of FBG (P=0.040), FIN (p<0.001), and HOMA-IR (p<0.001) and lower level of QUICKI (p<0.001) than the control individuals. The cases of PCOS-NIR had significantly lower levels of FIN (P=0.046) and HOMA-IR (P=0.039), and higher level of QUICKI (p<0.001) compared to the control group. The concentration of FBG had no significant variation in PCOS-NIR and control groups. As expected, the levels of FBG (p<0.001), FIN (p<0.001), and HOMA-IR (p<0.001) were significantly higher, and the level of QUICKI (p<0.001) was significantly lower in PCOS-IR subjects when compared to PCOS-NIR subjects.

ROC analyses revealed that the serum DPP4 activity could be a useful discriminator to distinguish PCOS-IR women and PCOS-NIR women from non-PCOS subjects (AUC 0.760, P=0.001, AUC 0.776, p<0.001, respectively). The cut-off value of DPP4 for PCOS-IR patients was found to be 2.23 and for PCOS-NIR patients was found to be 2.17 (table 2). 

**Table 1:** The demographic characteristics and biochemical variables in PCOS-NIR, PCOS-IR, and non-PCOS groups

**Variables**	**PCOS-NIR (N = 30)**	**PCOS-IR (N = 30)**	**non-PCOS (N = 30)**	**p-value**
Age (years)	29.20 ± 0.91	28.27 ± 0.90	29.73 ± 0.80	0.490
Weight (kg)	69.60 ± 2.54	70.20 ± 2.38	73.02 ± 1.94	0.537
BMI (kg/m^2^)	28.19 ± 1.01	29.17 ± 0.91	27.88 ± 0.88	0.597
Body fat%	35.14 ± 1.15	36.10 ± 1.22	34.76 ± 1.11	0.703
FBG(mg/dl)	75.75 ± 2.55	94.10 ± 3.37	83.76 ± 2.88	0.140^a^ 0.040^b^ < 0.001^c^
FIN(μIU/ml**)**	8.52 ± 0.48	20.89 ± 1.26	11.57 ± 0.74	0.046^a^ < 0.001^b^ < 0.001^c^
HOMA-IR	1.58 ± 0.09	4.82 ± 0.34	2.37 ± 0.15	0.039^a^ < 0.001^b^ < 0.001^c^
QUICKI	0.360 ± 0.004	0.306 ± 0.002	0.339 ± 0.003	< 0.001^a^ < 0.001^b^ < 0.001^c^
DPP4 activity **(**μM/mL/min**)**	2.43 ± 0.08	2.63 ± 0.16	1.91 ± 0.10	0.011^a^ < 0.001^b^ 0.511^c^
PGRN(ng/ml)	62.51 ± 13.33	63.94 ± 12.45	53.16 ± 11.41	0.259

BMI (Body mass index), FBG (Fasting blood glucose), FIN (Fasting insulin), HOMA-IR (Homeostatic model assessment of insulin resistance), QUICKI (Quantitative insulin sensitivity check index), DPP4 (Dipeptidyl peptidase-4), PGRN (Progranulin) Data are presented as means ± SE. **a**: PCOS-NIR vs. control, **b**: PCOS-IR vs. control, **c**: PCOS-NIR vs. PCOS-IR

**Table 2:** ROC curve analyses of DPP4 for discrimination of PCOS-IR and PCOS-NIR patients from non-PCOS women

**Group**	**Sensitivity**	**Specificity**	**AUC**	**95 % CI**	**p-value**
PCOS-NIR	%80	%76.7	0.776	0.647-0.904	< 0.001
PCOS-IR	%66.7	%80	0.760	0.638-0.882	0.001

AUC (area under the curve), CI (confidence interval). P < 0.05 is statistically significant

## Discussion

PCOS is identified as one of the causes of infertility in the female population; however, its pathogenesis is still unclear. This syndrome is often connected with IR and the increased risk of diabetes mellitus. IR and hyperinsulinemia are the momentous factors implicated in the development of PCOS (1). Adipose tissue in the role of an endocrine organ secretes several endocrine and paracrine factors called adipokines, which play critical impacts on the development of IR (21). In this study, we examined the changes in serum PGRN levels and DPP4 activity in PCOS women with and without IR, and non-PCOS controls. 

Recent studies have observed that the serum concentration of DPP4 increased in PCOS women. However, there are inconsistent results regarding the activity of DPP4 in PCOS subjects (10-12, 22). In our study, DPP4 activity significantly increased in PCOS-IR and PCOS-NIR patients compared to the non-PCOS group. Furthermore, the activity of this enzyme was higher in PCOS-IR than PCOS-NIR patients, without any significant difference between the two groups of PCOS. This finding suggests that the imbalance in the activity of this enzyme may be involved in the process of PCOS. Recently, evidence has suggested that DPP4 inhibitors may be a new treatment option in PCOS (23, 24). According to the results obtained from an animal study, DPP4 inhibitor sitagliptin treatment decreased FBG, testosterone level, and the ovarian fibrosis process in PCOS rats (23). Furthermore, a human study indicated that the administration of sitagliptin improved the function of beta-cell in obese PCOS cases with metformin intolerant (24). In line with our result, Blauschmidt et al. found that DPP4 serum activity was higher in PCOS patients than non-PCOS women. They also reported that an increase in androgens levels in vitro induced DPP4 gene expression. Interestingly, their results indicated a direct association between DPP4 activity and HOMA-IR (10). However, they did not separately investigate the activity of this enzyme in PCOS-IR and PCOS-NIR groups. In contrast to our findings, Braga et al. and Kahraman et al. displayed that the plasma and serum DPP4 activity was similar between healthy women and PCOS patients. Meanwhile, these studies found no association between the activity of DPP4 and FBG, FIN, and HOMA-IR in PCOS women (11, 12). There is no report regarding the connection between the activity of this enzyme and BMI in PCOS patients. In addition, a study after dividing individuals based on obesity found no change in serum activity of this enzyme between obese and non-obese PCOS groups, as well as between obese and non-obese controls (12). The explanation for the existence of contradictory results in relation to DPP4 activity in this syndrome may lie in the heterogeneity of PCOS cases. Moreover, the method of measuring enzyme activity may be partially involved in the inconsistent results of previous studies. The significant results in the study of Blauschmidt et al. may be influenced by sample size, which was more than the other studies. Hence, results may differ with a larger number of participants.

According to the ROC analyses, it seems that DPP4 activity has good diagnostic accuracy in distinguishing PCOS-IR (AUC 0.760) and PCOS-NIR (AUC 0.776) cases from non-PCOS women. Routinely, PCOS diagnosis in patients is based on Rotterdam criteria and there are not further accepted biomarkers for the PCOS diagnosis (10). Albeit, Dewailly et al. proposed that the serum level of anti-Müllerian hormone (AMH) could be included in the diagnostic criteria for PCOS (25). A recent investigation has demonstrated an association between the serum level of AMH and DPP4 activity in PCOS. They also compared the discrimination potential of AMH level (AUC = 0.827) with DPP4 (AUC = 0.609) for PCOS diagnosis and emphasized that DPP4 activity alone cannot be served as a biomarker (10). Further studies are required to investigate whether the combined analysis of serum DPP4 activity and other markers, including AMH, may enhance the probability of the prediction of PCOS. 

In this case-control study, we observed a tendency towards high of PGRN levels in the two groups of PCOS women than non-PCOS women; however, this variation was not significant. Moreover, its level was similar between the PCOS women with and without IR. Our finding negated this hypothesis that the PGRN level may be different in PCOS patients with and without IR, at least in this small population. Few studies have surveyed the relationship of PGRN level with PCOS regardless of the presence or absence of IR in the patients with PCOS (13, 26). In this regard, a study by Zhou et al. manifested that PGRN value in follicular fluid and its mRNA expression in granulosa cells in PCOS subjects was greater than the controls (13). Another study described an increase in circulating PGRN levels in subjects with PCOS (26). The sample size of the two mentioned studies was more than our study. Therefore, the no significant results may become statistically meaningful if larger samples were investigated. Ersoy et al. and Zhou et al. did not show any association between the levels of PGRN and BMI, FBG, FIN, and HOMA-IR in PCOS patients (13, 26). Although recent evidence has demonstrated that serum PGRN level is correlated with obesity in infertile women (27), and also in another study, its follicular fluid level was enhanced in overweight PCOS cases than normal-weight PCOS cases (13).

Nevertheless, why DPP4 activity and PGRN levels were not statistically different between PCOS-IR and PCOS-NIR cases are still unknown. Presumably, the results are influenced by sample size, clinical characteristics, and technical and analytical methods. There are limitations in our study that one of them is the small sample size. Only 90 women were enrolled in this case-control study. The other limitation is the measurement of the adipokines only in the serum of patients with PCOS. However, the advantage of our study is the investigation of DPP4 activity and PGRN levels in PCOS patients with and without IR. 

In conclusion, our study indicated that the serum activity of DPP4 was markedly increased in patients with PCOS-IR and PCOS-NIR compared to non-PCOS women. Hence, our study proposes that increasing the activity of this enzyme is presumably associated with PCOS. 

## Funding:

This study was financially supported by Babol University of Medical Sciences.

## Conflicts of interest:

The authors declare that there is no conflict of interest.
